# Transitions of care for people living with dementia – insights using linked data

**DOI:** 10.23889/ijpds.v9i5.2595

**Published:** 2024-09-18

**Authors:** Emma Gearon, Bronwyn Wyatt, Ingrid Evans, Megan Fraser, Ann-Kristin Raymer, Fleur de Crespigny, Melanie Dunford, Claire Sparke

**Affiliations:** 1 Australian Institute of Health and Welfare

## Objective

People living with dementia have increasing care needs as their condition progresses, and tend to be high users of health and aged care. Linked data provide an opportunity to better understand transitions between services, to enable quality and timely care.

## Approach

The pilot study used a new multi-source enduring linked dataset to analyse the use of health and residential aged care by Australians living with younger onset dementia between 2011–2016. The subsequent study focused on movements between hospital and residential aged care for older Australians who were hospitalised in 2017.

## Results

The younger onset dementia cohort comprised 5,400 Australians aged 30–69. More than half (58%) the cohort had moved from the community into residential aged care by the end of the study period. Of these, 21% had a hospital stay while living in the community that ended with entry to residential aged care. This important transition point was analysed further using a dementia cohort of 79,000 people aged 65 and over. One in 4 people (23%) with dementia who were living in the community moved into residential aged care within 7 days of a hospital stay. This group spent 20 days longer in hospital than other older people. People living in aged care were less likely than those in the community to return to hospital during the following year.

## Conclusions

These results can help inform planning of community-based dementia support services, improve coordination of care, and investigate sources of delay for people awaiting entry to aged care.

